# Methylation of the ribosomal RNA gene promoter is associated with aging and age‐related decline

**DOI:** 10.1111/acel.12603

**Published:** 2017-06-17

**Authors:** Patrizia D'Aquila, Alberto Montesanto, Maurizio Mandalà, Sabrina Garasto, Vincenzo Mari, Andrea Corsonello, Dina Bellizzi, Giuseppe Passarino

**Affiliations:** ^1^ Department of Biology, Ecology and Earth Sciences University of Calabria 87036 Rende Italy; ^2^ Italian National Research Center on Aging (INRCA) 87100 Cosenza Italy

**Keywords:** aging, biological age, chronological age, geriatric parameters, ribosomal DNA (rDNA) promoter, ribosomal RNA (rRNA) genes

## Abstract

The transcription of ribosomal RNA genes (rDNA) is subject to epigenetic regulation, as it is abrogated by the methylation of CpG dinucleotides within their promoter region. Here, we investigated, through Sequenom platform, the age‐related methylation status of the CpG island falling into the rDNA promoter in 472 blood samples from 20‐ to 105‐year‐old humans and in different tissues (blood, heart, liver, kidney, and testis) of 15 rats 3–96 weeks old. In humans, we did not find a consistently significant correlation between CpG site methylation and chronological age. Furthermore, the methylation levels of one of the analyzed CpG sites were negatively associated with both cognitive performance and survival chance measured in a 9‐year follow‐up study. We consistently confirmed such result in a replication sample. In rats, the analysis of the homologous region in the tissues revealed the existence of increased methylation in old rats. rRNA expression data, in both humans and rats, were consistent with observed methylation patterns, with a lower expression of rRNA in highly methylated samples. As chronological and biological ages in rats of a given strain are likely to be much closer to each other than in humans, these results seem to provide the first evidence that epigenetic modifications of rDNA change over time according to the aging decline. Thus, the methylation profile of rDNA may represent a potential biomarker of aging.

## Introduction

The transcription of nuclear ribosomal RNA (rRNA) genes is a key control point highly regulated in ribosome biogenesis which is crucial for cellular adaptation, stress response, cellular growth, and proliferation as well as to energetic requirements of cells (Russell & Zomerdijk, 2005; Grummt & Voit, 2010). In higher eukaryotes, a single transcription unit (~13 kb) contains the sequence for both the small (5.8S) and large (18S and 28S) rRNA molecules. Each unit is repeated in tandem and clusters of units are dispersed within nucleolar organizer regions and separated from each other by a nontranscribed spacer (~30 Kb) (Gonzalez & Sylvester, 1995). The mammalian rRNA gene promoter is composed by a core element spanning from −45 to +20 relative to the start site (+1), that is essential for accurate transcription initiation, and an upstream control element (UCE), from −180 to −107, displaying a regulatory role. Spacing between the two sequences and their relative orientation were demonstrated to be crucial for a correct functioning of the transcriptional apparatus (Paule & White, 2000). The promoter contains a number of short direct and inverted repeats and palindromes. By comparison of the regions in three mammalian species (human, mouse, and rat), several conserved sequences were found upstream and downstream the transcription starting point (Financsek *et al*., 1982).

rRNA genes are expressed in all tissues and, during development and differentiation, are subject to a strict regulation by a wide variety of factors including those belonging to JNK2‐MAPK‐mTOR, MYC, PKC, p53, and RAS/ERK pathways (Zhai & Comai, 2000; Mayer & Grummt, 2006; Song *et al*., 2013). Moreover, conditions supporting growth and proliferation, including nutrients, growth factors, glucose deprivation, and ATP levels, alter rDNA transcription mainly by modulating the activity of TIF‐IA factor (Grummt & Voit, 2010). In addition, several evidences demonstrated as rDNA expression is subject to epigenetic regulation in both physiological and pathological conditions (Grummt & Pikaard, 2003; Grummt, 2007). Mouse and human rDNA promoters show different CpG densities in the core promoter and UCE (Financsek *et al*., 1982). Promoters of active rRNA genes are devoid of CpG methylation and are associated with acetylated histones, whereas the reverse has been observed for silenced genes. In particular, DNA methyltransferase 1 (DNMT1) controls rDNA transcription and also plays a role in maintaining the nucleolar architecture (Majumder *et al*., 2006). In cultured mouse cells, rRNA synthesis has been observed to be regulated by the methylation of a single CpG dinucleotide located at ‐133 position within the UCE that inhibits the access of the RNA polymerase I transcription factor UBF (upstream binding factor) to the upstream control region of the promoter, thus abrogating rDNA transcription (Zatsepina *et al*., 1993; Ghoshal *et al*., 2004). In humans, it was reported the lacking of methylation within the transcriptionally active rDNA promoter, whereas this epigenetic modification seems to be a hallmark of the transcriptionally inactive fraction. Significant hypomethylation of the rDNA promoter was observed in human hepatocellular carcinoma samples, with consistent high level of rRNA synthesis in rapidly proliferating tumors as well as in prostate cancer cell lines, although the hypomethylation observed was not linked to rRNA overexpression (Ghoshal *et al*., 2004; Uemura *et al*., 2012). Conversely, extensive methylation patterns in patients with breast and endometrial carcinoma have been detected (Yan *et al*., 2000; Powell *et al*., 2002). In the latter, differences in DNA methylation were observed between African American and Caucasian patients.

Tissue‐specific hypermethylation of rDNA promoter has also been correlated with Alzheimer's disease, resulting in the silencing of the nucleolar chromatin and, in turn, in the alteration of the ribosomal biogenesis and protein synthesis (Pietrzak *et al*., 2011).

In the field of aging epigenetics, it has emerged an age‐related increase in DNA methylation at the 5′ end of the 18S and 28S ribosomal RNA genes in several tissues of differently aged mice as well as in sperm and liver of male rats (Swisshelm *et al*., 1990; Oakes *et al*., 2003). In addition, *in vitro* senescence of human fibroblasts from both normal donors and Werner syndrome patients was accompanied by high methylation levels of the RNA repeats (Machwe *et al*., 2000). Conversely, a study carried out on young and old murine hematopoietic stem cells (HSCs) indicated a hypomethylation of rRNA genes with their consequent increased transcription (Sun *et al*., 2014).

We investigated the potential association of methylation levels of the rDNA promoter with human aging in an elderly population. These levels were measured by applying the Sequenom EpiTYPER platform to peripheral blood DNAs collected from 472 subjects from Southern Italy (Calabria) and in two replication samples including 296 subjects. In order to assess an evolutionary conservation of the methylation patterns and to understand variation between and within tissues across aging, we also replicated the study in differently aged rats in which the methylation levels of the homologous promoter region were determined in peripheral blood, heart, liver, kidney, and testis. In addition, the functional relevance of the observed methylation patterns was explored by measuring the rRNA expression in differently aged human and rat samples.

## Results

### CpG methylation profiling of ribosomal RNA gene promoter in differently aged humans

**Figure 1:**
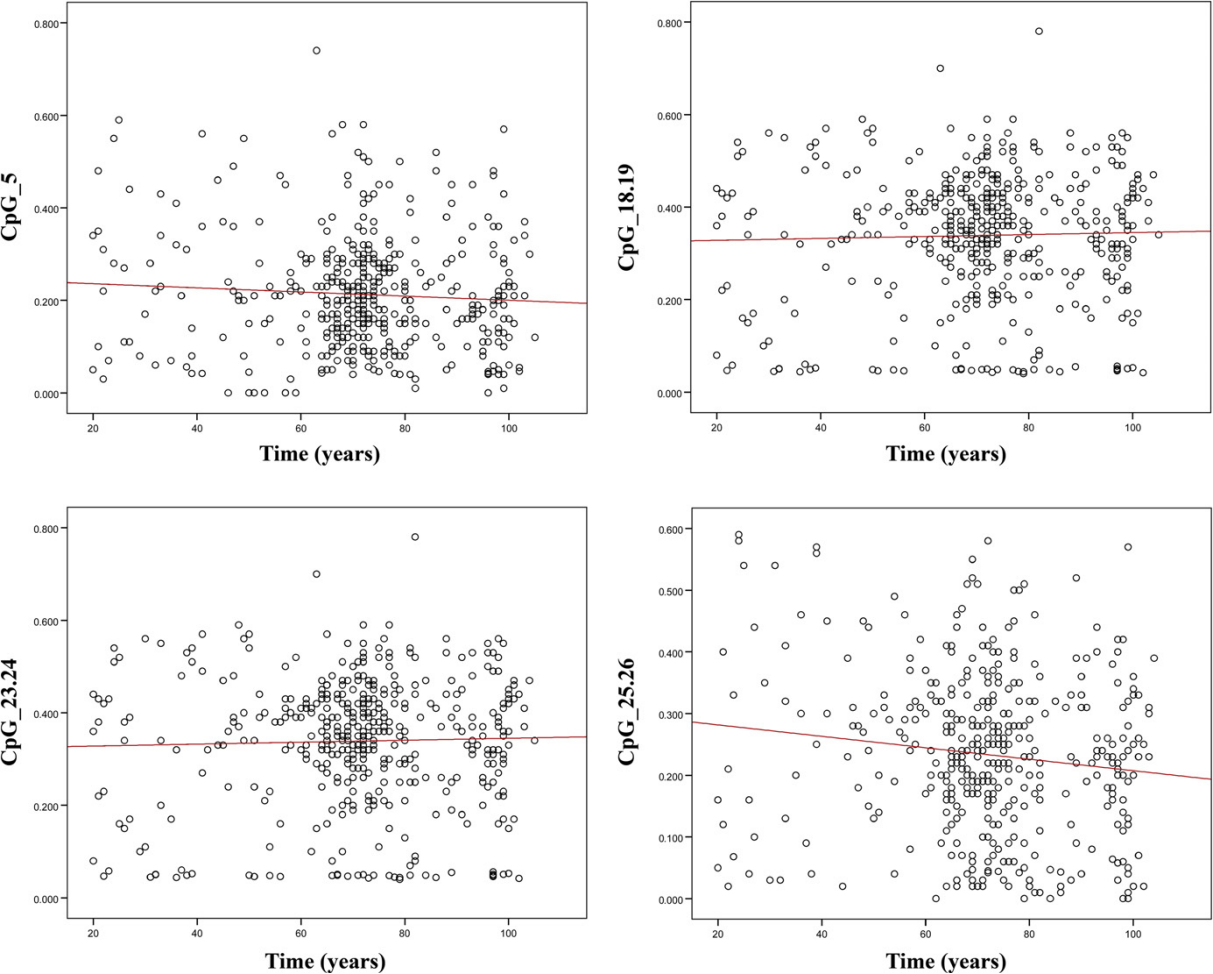
Scatter plot of DNA methylation values as a function of human age. For each CpG unit, the red straight line represents least‐squares linear regression line.

We adopted Sequenom MassARRAY EpiTYPER, a bisulfite‐based technology that relies on base‐specific cleavage and mass spectrometry, to measure the methylation levels of CpG dinucleotides located within different *cis* elements (spanning from −189 to +47 bp according to the ATG position) in the human promoter of the rRNA genes. In particular, a CpG island, encompassing 19 CpGs in the upstream control element (UCE) and 8 CpGs in the core promoter, was analyzed (Fig. S1, Supporting information). Bioinformatics analysis also revealed the presence of several transcription factors binding sites (Fig. S1, Supporting information). The 27 CpG residues were assayed as 11 Sequenom analytic units containing one (2 units), two (6 units), three (2 unit), or seven (1 unit) individual CpG sites (Fig. S1, Supporting information). DNA methylation was investigated in bisulfite‐treated DNA samples collected from 472 subjects (20‐ to 105‐year‐old subjects) of the discovery dataset to assess potential epigenetic changes during aging. After quality control, only the units CpG_5, CpG_18.19, CpG_23.24, and CpG_25.26 were considered in the analysis. Figure 1 shows the methylation values for the four CpG units by age group. A significant decrease with age in the methylation level of CpG_25.26 emerges (*P* = 0.008), while no significant difference can be observed for the other CpG units. In addition, no significant change was observed between men and women for each analyzed unit (data not shown). However, no significant association was detected between methylation changes and age for any of the four CpG units analyzed in the 296 samples of the replication study (RD1 and RD2).

### Correlation of the rDNA methylation profiles with geriatric components and survival at old age

We verified whether the variability of methylation of the rDNA promoter might affect the quality of life in the 60‐ to 89‐year‐old group of the discovery dataset. In particular, this age group was analyzed for examining the association between the methylation level variability at the analyzed CpG units and physical and cognitive abilities measured by geriatric assessments, including Hand Grip (HG), Mini Mental State Examination (MMSE), Activity Daily Living (ADL) and Geriatric Depression Scale (GDS). As shown in Table 1, subjects with higher methylation levels at CpG_5 unit showed an impaired cognitive performance (*P *= 0.039). In addition, a borderline negative correlation was also observed between CpG_25.26 methylation variability and functional status (*P* = 0.064). Conversely, no significant association was observed between methylation levels of the promoter region of the rRNA genes and the remaining geriatric parameters.

**Table 1:** Mean methylation values at the analyzed CpG units (standard error in parenthesis) with respect to physical, cognitive, and functional performances in the discovery dataset. For each geriatric parameter, the compared groups were obtained using the cutoff defined in the Experimental procedures section. For Hand Grip (HG), the third quartile of the relevant frequency distribution was used to identify subjects with a good (Q4) or normal/poor (Q1‐Q3) performance; for Mini Mental State Examination (MMSE), a score of 23 was considered as cutoff for recognizing impaired or conserved cognitive functioning; for Activity of Daily Living (ADL), a score of 5 was used to define a subject as fully independent in the tested activities; for Geriatric Depression Scale (GDS), a total score of 5 was considered a cutoff for recognizing subjects showing depression symptoms

CpG unit	HG			MMSE			ADL			GDS		
	Q1‐Q3	Q4	*P* a	≥23 (*N* = 115)	<23 (*N* = 87)	*P* a	No disability (*N* = 162)	At least 1 disability (*N* = 30)	*P* a	GDS>5	GDS<=5	*P* a
CpG_5	0.21 (0.01)	0.23 (0.01)	0.464	0.20 (0.01)	0.23 (0.01)	0.039	0.22 (0.01)	0.21 (0.02)	0.690	0.21 (0.01)	0.22 (0.01)	0.398
CpG_18.19	0.34 (0.02)	0.37 (0.02)	0.098	0.35 (0.01)	0.33 (0.02)	0.319	0.35 (0.01)	0.33 (0.03)	0.421	0.34 (0.01)	0.35 (0.01)	0.568
CpG_23.24	0.34 (0.02)	0.37 (0.02)	0.099	0.35 (0.01)	0.33 (0.02)	0.323	0.35 (0.01)	0.33 (0.03)	0.425	0.34 (0.01)	0.35 (0.01)	0.582
CpG_25.26	0.22 (0.01)	0.23 (0.01)	0.547	0.23 (0.01)	0.21 (0.01)	0.247	0.23 (0.01)	0.19 (0.03)	0.064	0.22 (0.01)	0.23 (0.01)	0.649

a *P*‐value refers to the *t*‐test for independent samples.

To further confirm the association between methylation levels of CpG_5 unit and cognitive performance, we analyzed the methylation levels at this unit with two additional tests of episodic memory (recall and repeat test, see Experimental procedures). Once again, we found a significant negative correlation between methylation levels at CpG_5 unit and the number of repeated words in the recall test (*r* = −0.128, *P* = 0.036). Conversely, no significant association was detected for the number of repeated words in the repeat test (*r* = −0.135, *P* = 0.071).

**Figure 2:**
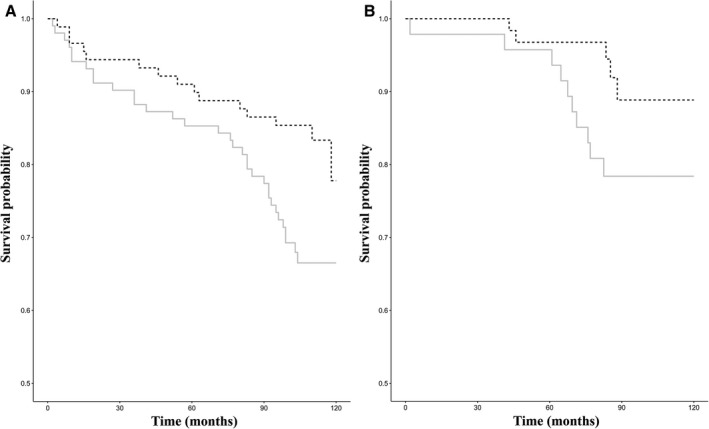
Kaplan–Meier survival curves of human samples stratified according to CpG_5 methylation status of samples belonging to the discovery (A) and replication (B) datasets. Solid line represents the subgroup of subjects with a methylation level higher than 20%; dashed line represents the subgroup with a methylation level lower than (or equal to) 20%.

Finally, to better evaluate the relationship between DNA methylation levels and survival at older ages, we compared the survival curves of subjects that exhibited low methylation levels with those showing high methylation levels at the baseline visit in the discovery dataset. In the 60‐ to 89‐year‐old group, we found that subjects displaying a high level of methylation at the CpG_5 unit had a significantly reduced survival chance (*P* = 0.012) (Fig. 2A). Indeed, after adjusting for age and gender, the mortality risk of subjects with a methylation level higher than 20% was about twofold increased with respect to the subjects with low methylation levels (HR = 2.073; *P* = 0.0020). On the contrary, methylation levels at the remaining CpG units did not affect survival chances in the analyzed follow‐up period. In the 90+‐year‐old group, no association was detected between methylation variability and survival at old ages. It is worth to note that after the Bonferroni's correction, the association between the CpG_5 unit and survival remained statistically significant.

By comparing the survival curves of subjects that exhibited low methylation with those showing high methylation levels in the RD1 dataset, we successfully replicated the association between CpG_5 methylation variability and survival in the follow‐up period. In particular, we found that subjects displaying a high level of methylation at the CpG_5 unit (>20%) had a significantly reduced survival chance (*P* = 0.020) (Fig. 2B). Also in this case, after adjusting for age and gender, the mortality risk of subjects with a methylation level higher than 20% was about 3.5‐fold increased with respect to the subjects with low methylation levels (HR = 3.54; *P* = 0.022).

### CpG methylation profiling of ribosomal RNA gene promoter in rats

**Figure 3:**
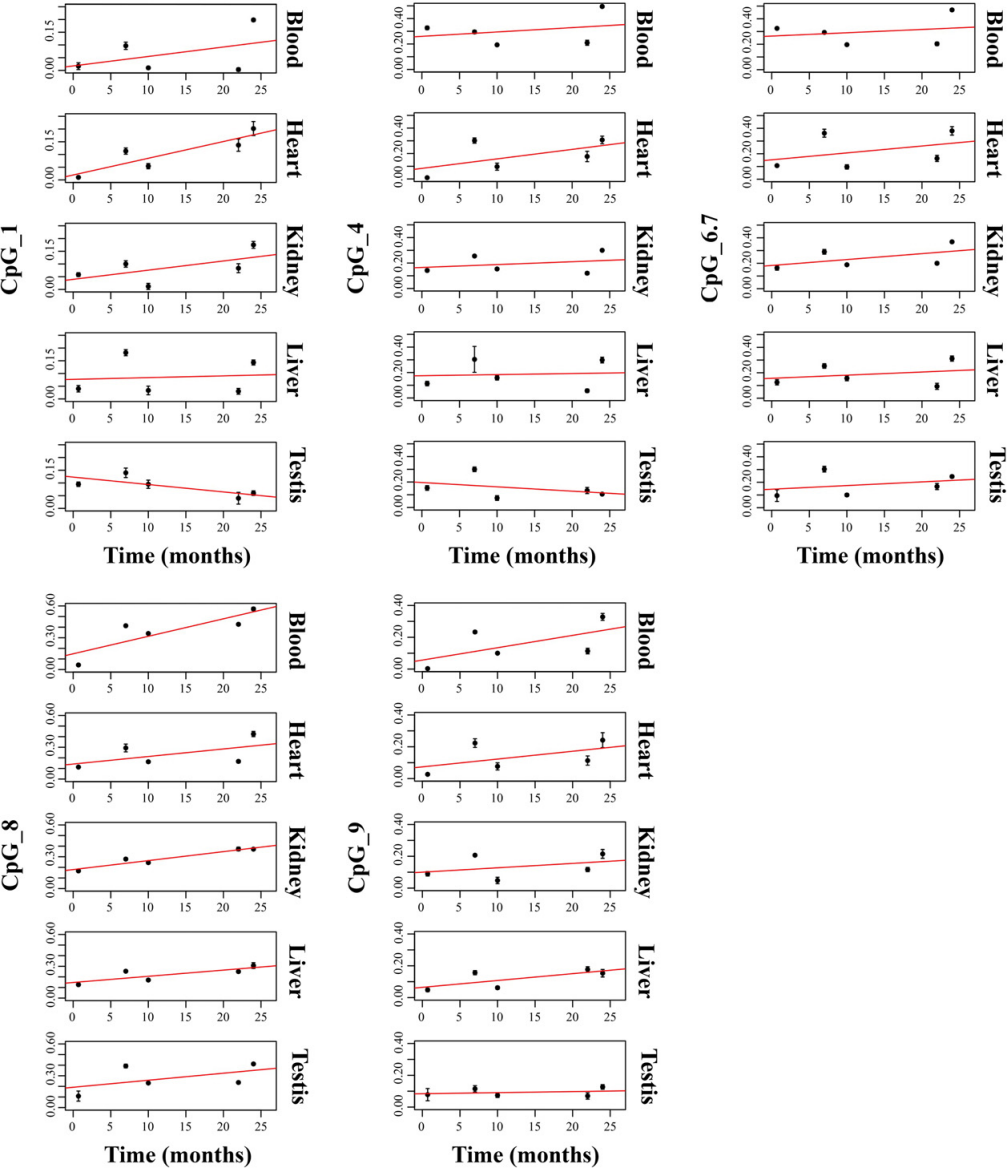
Scatter plot of DNA methylation values as a function of age in rats. For each CpG unit and tissue, the red straight line represents least‐squares linear regression line. Values are reported as the mean of three independent triplicate experiments carried out for each time point.

Methylation levels were also evaluated in the rat rDNA promoter. In particular, a region ranging from −193 to +40 bp, according to the ATG position and containing homologous nucleotides to the human sequence previously described, was analyzed (Fig. S2, Supporting information). It encompasses 9 CpGs assayed as 6 Sequenom analytic units containing one (4 units) and two (2 units) individual CpG sites (Fig. S2, Supporting information). Bioinformatics analysis also revealed the presence of several transcription factors binding sites (Fig. S2, Supporting information). DNA methylation was investigated in bisulfite‐treated DNA samples extracted from blood, heart, liver, kidney, and testis of differently aged rats (3, 28, 40, 88, and 96 weeks old). As result from quality control process, only the units CpG_1, CpG_4, CpG_6.7, CpG_8, and CpG_9 were considered in the analysis. Figure S3 (Supporting information) shows that the tissue‐specific methylation levels of most of the CpG units we analyzed exhibit a peculiar trend. In particular, a linear regression analysis of the methylation levels as a function of age revealed that in some CpG units, a general increase occurred, regardless of the tissue type (Fig. 3). In fact, methylation levels at CpG_1 and at CpG_8 significantly increase in heart and in kidney (*P* = 0.010), respectively. A borderline significant increase was observed for CpG_8 in blood (*P* = 0.074) and liver (*P* = 0.097). In addition, it should be noticed that the highest R‐square values were observed for CpG_8 in kidney, blood, and liver (0.920, 0.708, 0.654, respectively). In particular, the most important variation has been detected in blood, where an about tenfold increase has been observed in the analyzed time period. Finally, no significant difference in methylation levels was observed in testis.

### Expression of rRNA in human and rat samples

**Figure 4:**
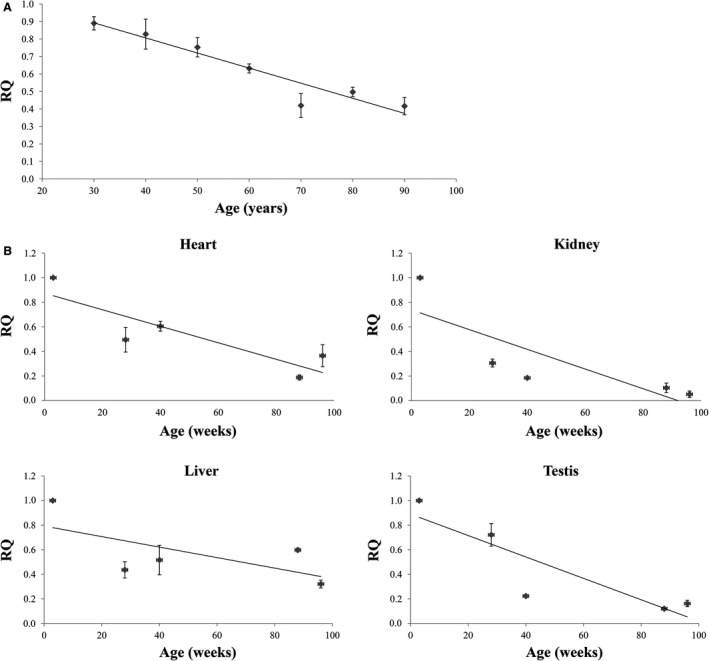
Expression levels of rDNA measured in human blood (A) and heart, liver, kidney, and testis from rats (B) of different aged. These levels are reported as the mean of relative quantification values (RQ), measured in three independent triplicate experiments with standard error mean (SEM).

To explore the functional relevance of the methylation of the CpG island located within the rDNA promoter, quantitative real‐time PCR assays were carried out to evaluate the expression levels of rRNA in samples of different age from human blood as well as from heart, liver, kidney, and testis of rats. In both human and rat samples, we observed that rRNA levels decrease late in life (Fig. 4A,B). More specifically, with respect to the thirties, a decline in the rRNA expression, which confirms the suppressive role of DNA methylation on transcription, was observed in humans after 60 years (*P* < 0.004).

## Discussion

Alteration of the ribosome biogenesis and an overall protein synthesis rate decline have been observed to characterize aging process in many organisms, including humans (Syntichaki *et al*., 2007; Tavernarakis, 2008; Charmpilas *et al*., 2015). This decline could be an effect of the progressive deterioration in most cellular functions usually associated with aging, or it could be a concurrent factor in the process. If to date a general reduction of protein synthesis has been attributed to the decreased frequency of mRNA translation, current studies, reporting an involvement of epigenetic mechanisms in silencing a large fraction of the rRNA genes, with a consequent impairment of rDNA function, could lead to a new understanding of the phenomenon (Syntichaki *et al*., 2007; Hands *et al*., 2009).

On the basis of these evidences, we investigated whether changes in the DNA methylation patterns of the rRNA gene promoter take place during the lifetime. DNA samples were extracted from whole blood collected from differently aged human individuals displaying different phenotypes according to cognitive, functional, and psychological parameters (Montesanto *et al*., 2010). We did not find a consistent statistically significant association between the methylation levels of the analyzed CpG sites with the age of the donor, although a slight association had been suggested by the first dataset. In fact, multiple test corrections and a replication dataset did not confirm this observation.

On the other hand, although it is not associated with chronological aging, in middle/advanced‐aged subjects the variability of CpG_5 methylation was found to be significantly correlated with both cognitive performances and survival in the 9‐year follow‐up period. This last result, which held multiple test correction, was further confirmed in the replication sample. These results are consistent with previous data demonstrating that changes in the methylation levels of a single or a few CpG sites have been associated to complex phenotypes, because they are probably located in specific functional regions; in most cases, including gastric and colon cancer, rheumatoid arthritis and type 1 diabetes, these findings were always related to the regulation of the associated gene (Joensuu *et al*., 2015; Yara *et al*., 2015; Zhao *et al*., 2015). Our results seem to be particularly attractive, because they show a fine remodeling of the methylation profile associated with the biological aging rather than to the chronological age and contribute to better clarify the molecular basis of the interindividual psychophysical vulnerability that characterizes the old age (Liu *et al*., 2009; Bellizzi *et al*., 2012). The effects at molecular levels of the above association have to be clarified, but it is plausible to hypothesize that the decrease in the rRNA levels we observed late in life may be determined by the methylation of the CpG_5 site that in turn might be driven by multiple factors, including genetic variations, diet, environment, and the interindividual variation of the structure of rDNA cluster itself (Caburet *et al*., 2005; Stults *et al*., 2008; Zampieri *et al*., 2010). The careful attention to the quality of the sampling and the possibility to test the obtained results in an independent sample, in which the association between the methylation level of CpG_5 site with survival was consistently replicated, lead us to generalize the findings reported and to consider this site a potential biomarker of the functional decline typical of aging.

The study we carried out in rats demonstrated the presence of methylated CpGs within the rDNA promoter, which are also located in regions highly conserved during the evolution (Financsek *et al*., 1982). Changes we observed in the methylation patterns occurring during lifetime in these organisms have a functional relevance as demonstrated by the decrease in rRNA levels we observed in almost all the tissue analyzed.

It is plausible to assume that the greater concordance of results between biological and chronological age in rats is due their lower interindividual variability, because they are reared in standard animal house and devoid to variation in environmental factors. Conversely, multiple factors, such as genetic and environmental variations, may contribute to widening the gap between the chronological and biological aging in human, by introducing confounding elements in the determination of qualitatively heterogeneous aging phenotypes regardless of the chronological age, especially late in life, as previously observed (Bellizzi *et al*., 2012). Further investigations might better clarify this difference, especially considering that the two analyzed regions are recognition target for different transcription factors whose regulation, together with the different methylation patterns, could have specie‐specific functional impacts.

Notwithstanding that, the progressive age‐related increase in the methylation levels of the most CpG sites in the rats bears a resemblance to the increase in methylation levels of CpG_5 associated in humans with the biological aging. How could the methylation changes of peculiar CpG sites be functionally involved in the functional decline characterizing the aging process? If the epigenetic modification of functional sites may hamper ribosomal biogenesis, this may drastically reduce the cellular protein synthesis, being ultimately responsible of those multisystem deficits occurring over the lifetime. Thus, an interdependence seems to exist between rDNA promoter methylation and the aging process, and in particular with the aging associated decay, and these sites may represent an potential evolutionary conserved biomarker of the rate of the aging process.

The analysis of DNA samples extracted from peripheral whole blood for epigenetic studies is currently debated due to the cellular heterogeneity of white blood cells, that, in combination with changes in subtype composition occurring in physiological (see aging) and pathological conditions and cell specific methylation profiles, could compromise DNA methylation measurements (Adalsteinsson *et al*., 2012; Yuan *et al*., 2015). In this context, data we observed in rats can contribute to the above debate because the DNA methylation profiles in different tissues displayed comparable trends, suggesting that the composition of different blood cell subtypes should not substantially affect the overall outcome and, thus, the blood can be considered as a representative model.

This study has clearly demonstrated that promoter region of rRNA genes is epigenetically regulated throughout life in human and model organisms. In particular, this regulation has a major impact at biological than chronological age as the methylation levels of the CpG_5 site were consistently associated with life expectancy in two independent samples. This evidence demonstrates once again the significant impact that epigenetics have on the progressive decline defining and characterizing aging process. rDNA methylation could represent new challenge in the characterization of the biological hallmarks of aging, contributing to clarify the molecular basis of gene expression remodeling and the variety of phenotypes occurring during lifetime. Moreover, the reversibility of the epigenetic marks could prompt the search for therapeutic agents able to slow down the above decline likely due to the epigenetic deregulation.

## Experimental procedures

### Population samples

The discovery dataset included 472 unrelated individuals (224 men end 248 women) with a median age of 72 years. The recruitment of subjects older than 90 years was carried out between 2002 and 2005 through the population registries of Calabrian municipalities. Subjects in the age range 60–89 years (*n* = 264) were recruited between 2004 and 2007 as part of a survey aimed at monitoring the health status of this population segment in Calabria. These subjects underwent through a geriatric assessment and a structured interview including the administration of a questionnaire validated at European level. The questionnaire collected socio‐demographic information, anthropometric measures and a set of the most common tests to assess cognitive functioning, functional activity, physical performance, and depression. In addition, common clinical hematological tests were performed.

The analyses were carried out by dividing the sample in three specific age classes obtained according to the survival function of the Italian population from 1890 onward (Passarino *et al*., 2006). The two age ‘thresholds’ used to define these age classes were 60 and 89 years, respectively. The first cutoff corresponds to the point after which we observe a significant negative change in the slope of the survival curve of the Italian population. In particular, the first age group now included 88 subjects younger than 60 years; the second age group included 296 60‐ to 89‐year‐old subjects; finally, the third age group included 88 subjects older than 89 years.

Vital status for 294 subjects (99.3%) of the 60‐ to 89‐year‐old group and for 88 subjects (100%) of the oldest group was traced after about a mean follow‐up time of 7 and 9 years, respectively, through the population registers of the municipalities where the respondents lived.

Two replication samples included 296 subjects (155 females and 141 males) were recruited at the INRCA Hospital, which is a reference point for the care of the aging people in the Calabria. The first replication dataset (RD1) included 128 subjects (73 females and 55 males) whose ages ranged between 50 and 75 years. Vital status for 110 subjects (85.9%) was traced after about a mean follow‐up time of 8 years through the population registers of the municipalities where the respondents lived.

The second replication dataset (RD2) included 168 subjects (82 females and 86 males) whose ages ranged between 18 and 93 years in the frame of an ongoing research project aimed at identifying the epigenetic signature of the aging process.

Fully informed consent was obtained in writing from all the participants, and all the studies were approved by the Local Ethics Committee.

### Geriatric assessment

Hand Grip (HG) strength was measured using a handheld dynamometer (SMEDLEY's dynamometer TTM), while the subject was sitting with the arm close to his/her body. The test was repeated three times with the stronger hand. The maximum of these values was used in the analyses. As HG strength is affected by age, sex and height the scores were corrected for these factors. When a test was not carried out, it was specified if it was due to physical disabilities or because the subject refused to participate.

Mini Mental State Examination (MMSE) test was used to evaluate the cognitive performance in the analyzed sample. It is a 30‐item questionnaire that assesses orientation, episodic memory, attention, language, and construction functions (Folstein *et al*., 1975). As the test is affected by age and educational status, the MMSE scores were normalized for these variables according to a standardized procedure (Grigoletto *et al*., 1999). A score of 23 was considered as cutoff for recognizing impaired or conserved cognitive functioning.

The management of Activities of Daily Living (ADL), such as bathing, dressing, toileting, transfer from bed to chair, and feeding, was assessed using a modification of the Katz’ Index of ADL (Katz *et al*., 1970). The assessment was based on what the subject was able to do at the time of the visit. The score is given counting the number of activities in which the participant is dependent or independent at the time of the visit. For the following analyses, ADL scores were dichotomized as 1 if the subject was independent in all items and 0 otherwise.

Episodic memory was evaluated by two tests of immediate and delayed recall of a 12‐word list (McGue & Christensen, 2001; Nilsson *et al*., 2006). The interviewer read 12 words to the subject. Then, the subject was invited to repeat all the words he/she caught (Recognition and Registration test). After a few minutes where the subject was distracted with other questions, the interviewer asked the subject to repeat all the words he/she could remember of the 12‐word list (Delayed Recall test).

The Geriatric Depression Scale (GDS) is an instrument designed to measure depression in the elderly consisting of a series of yes/no questions in reference to how they felt on the day of administration. A score above 5 is suggestive of moderate depression, while a score above 10 is suggestive of severe depression. For the following analyses, GDS scores were dichotomized as 1 if the subject showed depression symptoms (GDS>5) and 0 otherwise.

### Animals

Experiments were performed on 3‐, 28‐, 40‐, 88‐, and 96‐week‐old Sprague Dawley rats (*n* = 3 for each age group) breeding locally in the animal care of the University of Calabria (Italy). Animals were housed in light (12:12‐h light–dark cycle) and temperature (22 °C)‐controlled rooms with free access to food (ssniff diet V1535, German, metabolizable energy 3.057 Kcal kg^−1^) and water. Animals were euthanized with inhalation of diethyl ether followed by cervical transection. All procedures were conducted according to the European Guidelines for the care and use of laboratory animals (Directive 2010/63/EU) and in accordance with Italian law.

### DNA samples

Six milliliters of venous blood was drawn from each human subject. Plasma/sera were used for routine laboratory analyses, while DNA was extracted from buffy coats following standard procedures.

500 μL of rat peripheral blood was drawn by cardiac puncture and kept on ice in presence of DNA extraction buffer (10 mm NaCl, 20 mm Tris–HCl pH 8.0, 1 mm EDTA). Heart, liver, kidney, and testis were excised, placed in cold HEPES‐physiological saline solution (HEPES‐PSS), weighed, and thoroughly homogenized in the presence of DNA extraction buffer. Then, 10% SDS and 10 mg mL^−1^ of proteinase K were added to all samples which were vigorously vortexed and incubated at 37 °C for 48 h with periodical mixing. Genomic DNA was obtained by phenol/chloroform purification. The DNA concentration and purity were determined spectrophotometrically.

### Primer design for EpiTYPER assay

The region of 239 bp of the human rRNA promoter harboring the CpG island was amplified using the following primers: RibFor 5′‐AGGTTTTTGGGTTGATTAGA‐3′ and RibRev 5′‐AAAACCCAACCTCTCC‐3′. The region of 263 bp of the rat rRNA promoter harboring the CpG island was amplified using the following primers: Rat For 5′‐GGTTTGTGAGTATTTAGGGTTTTAAGG‐3′ and Rat Rev 5′‐CAACCTTAATACAAACCTCTT

CCAA‐3′. A T7‐promoter tag (CAGTAATACGACTCACTATAGGGAGAAGGCT) was added to the reverse primers for the *in vitro* T7 transcription, and a 10‐mer tag sequence (AGGAAGAGAG) was added to the forward primers to balance the PCR primer length.

### Bisulfite treatment and PCR conditions

Bisulfite conversion of each DNA sample was performed using EZ‐96 DNA Methylation‐Gold kit (Zymo Research, Euroclone, Milan, Italy), according to the manufacturer's protocol. Briefly, 1 μg of genomic DNA was added to 130 μL of CT conversion reagent in a final volume of 150 μL. The mix was incubated at 98 °C for 10 min and, successively, at 64 °C for 2.5 h. After adding 400 μL of M‐binding buffer to the wells of the silicon‐A binding plate, each sample was loaded into the wells and centrifuged at 3000 *g* for 5 min. After adding of 400 μL of M‐wash buffer to the wells and a centrifugation at 3000 *g* for 5 min, 200 μL of M‐desulfonation buffer was added to each well and incubated at room temperature for 20 min. Then, the solution was removed by centrifugation at 3000 *g* for 5 min and the wells were washed twice with 400 μL of M‐wash buffer. Deaminated DNA was eluted in 30 μL of M‐elution buffer.

The PCRs were carried out in a total volume of 5 μL using 1 μL of bisulfite‐treated DNA, EpiTaq PCR buffer 1X, 0.4 μm of each primer, 0.3 mm dNTP mixture, 2.5 mm of MgCl_2,_ and 0.005 U TaKaRa EpiTaq HS (TaKaRa, Diatech Lab Line, Milan, Italy). The thermal profile used for the reaction included a 4‐min heat activation of the enzyme at 95 °C, followed by 45 cycles of denaturation at 94 °C for 20 s, annealing at 60 °C for 30 s, extension at 72 °C for 1 min, then one cycle at 72 °C for 3 min. 0.5 μL of each PCR product was electrophoresed on 1.5% agarose gel to confirm successful PCR amplification and amplification specificity.

### Dephosphorylation of unincorporated deoxynucleotide triphosphates and *in vitro* transcription and RNaseA cleavage

Unincorporated dNTPs in the amplification products were dephosphorylated by adding 1.7 μL Dnase free water and 0.3 μL (0.5 U) shrimp alkaline phosphatase (SAP) (Sequenom, Inc., San Diego, CA, USA). Each reaction was incubated at 37 °C for 40 min, and SAP was then heat inactivated for 5 min at 85 °C. Subsequently, samples were incubated for 3 h at 37 °C with 5 μL of T‐cleavage reaction mix (Sequenom), containing 3.21 μL RNAse‐free water, 0.89 μL 5× T7 polymerase buffer, 0.22 μL T‐cleavage mix, 0.22 μL 100 mm DTT, 0.40 μL T7 RNA & DNA polymerase and 0.06 μL RNAse A, for concurrent *in vitro* transcription and base‐specific cleavage. The samples of cleaved fragments were then diluted with 20 μL water. Conditioning of the cleavage reaction was carried out by adding 6 mg of clean resin.

### Mass spectrometry

10 nL of the resultant cleavage reactions was spotted onto silicon matrix‐preloaded chips (Spectro‐CHIP; Sequenom) using a MassARRAY nanodispenser (Sequenom) and analyzed using the MassARRAY Compact System matrix‐assisted laser desorption/ionization‐time‐of‐flight mass spectrometer (MALDI‐TOF) (Sequenom). The spectra's methylation ratios were calculated using epityper software v1.0 (Sequenom). The method yields quantitative results for each of the sequence‐defined analytic units referred as CpG units, which may contain either one individual CpG site or an aggregate of downstream CpG sites. Triplicate independent analyses from sodium bisulfite‐treated DNA sample were undertaken.

The effectiveness of the entire experimental procedure was assayed by analyzing as control CpGenome Universal Unmethylated DNA (Chemicon) and CpGenome Universal Methylated DNA (Chemicon, Millipore, Germany) in serial mixtures of methylated and unmethylated products, with 10% methylation increments.

Data quality control and filtering were carried out by the removal of the CpG dinucleotides whose the measurement success rate was <80%. Poor‐quality and nonvaluable data for the quantitative methylation of each CpG unit measured by MALDI‐TOF‐MS were excluded.

### Expression profile analysis of rRNA in human and rats

Total RNA extracted from the blood of individuals of various age (30–90 years old) using ReliaPrep RNA Tissue Miniprep System (PromegaCorp, Italy). Briefly, 2.5 mL of fresh whole blood was mixed to 7.5 mL of RNA red blood cell lysis solution and incubated for 10 min at room temperature. White cells were isolated by centrifugating samples at 3000 *g* for 10 min and lysed in 200 μL of LBA+TG buffer and 85% μL of isopropanol. The lysate was then transferred to a ReliaPrep minicolumn, and RNA was purified according manufacturer's recommendation.

Total RNA from frozen heart, liver, kidney, and testis was homogenized in buffer RTL and purified using RNeasy Mini Kit (Qiagen, Milan, Italy) according manufacturer's recommendations. RNA concentration was measured for each sample using a spectrophotometer and purity of the sample evaluated using the 260/280 nm absorbance ratio. RNA samples were treated with DNA‐free DNase to remove any residual genomic DNA contamination.

Reverse transcriptase‐PCRs (RT–PCR) were carried out using the RevertAid RT Kit (Thermo Fisher Scientific, Milan, italy). First, a RT mix including 500 ng of total RNA and 1 μL of Oligo(dT)_18_ primers was preheated at 65 °C for 5 min. Then, the reaction was carried out in a 20 μL final volume containing 1 × reaction buffer, 20 U of RiboLock RNase inhibitor, 1 mm of dNTP mix, and 200 U of RevertAid M‐MuLV RT reverse transcriptase. The mix was incubated at for 60 min at 42 °C and, successively, at 70 °C for 5 min to inactivate the reverse transcriptase. The cDNAs obtained were then used as a template for real‐time PCRs carried out using the SYBR Green qPCR Master Mix (Promega) in a StepOne Plus machine (Applied Biosystems, Milan, Italy).

Forward and reverse primers used for human (h) and rat (r) samples were as follows: hGAPDH For 5′‐ATGGGGAAGGTGAAGGTCG‐3′; hGAPDH Rev 5′‐GGGGTCATTGATGGCAACAATA‐3′; hrRNA For 5′‐GAACGGTGGTGTGTCGTT‐3′; hrRNA Rev 5′‐GCGTCTCGTCTCGTCTCACT‐3′; rGAPDH For 5′‐CCCCCAATGTATCCGTTGTG‐3′; rGAPDH Rev 5′‐TAGCCCAGGATGCCCTTTAGT‐3′; rrRNA For 5′‐TCGAACTTGACTATCTAGAGG‐3′; rrRNA Rev 5′‐GAAGGAGGAACACTCACAC‐3′. The final PCR mixture (10 μL) contained 1 μL of cDNA, 1× GoTaq^®^ qPCR Master Mix, 0.2 μm of each primer, and 1× CXR reference dye. The thermal profile used for the reaction included a 2‐min heat activation of the enzyme at 95 °C, followed by 35 cycles of denaturation at 95 °C for 15 s and annealing/extension at 60 °C for 60 s, followed by melt analysis ramping at 60–95 °C. All measurements were taken in the log phase of amplification. Negative controls (in which water instead of cDNA was added) were also run in each plate. stepone Software V 2.0 was used to analyze data. Gene expression values were normalized to GAPDH gene expression, used as internal control. In addition, the normalized values measured in the 30‐year‐ and 3 week‐old human and rat samples, respectively, were used as reference values (relative quantification) for the other samples.

### Statistical analysis

Linear regression analyses were carried out to evaluate the effect of DNA methylation values as a function of age. Student's *t*‐test was adopted to compare methylation profiles with respect to the analyzed geriatric parameters. To this purpose, GDS scores were dichotomized as one if the subject showed depression symptoms (GDS>5) and zero otherwise, because a total score higher than 5 is suggested as a benchmark to indicate depression (Fountoulakis *et al*., 1999); MMSE scores were dichotomized as 1 if the subject showed a normal cognitive function (MMSE >23) and zero otherwise, because a score of 23 is a generally accepted cutoff score for recognizing impaired or conserved cognitive functioning (Lopez *et al*., 2005). ADL scores were dichotomized as one if the subject was not independent in all five items and zero otherwise, because disability was defined as the inability to perform at least one ADL without using personal assistance or special equipment. Finally, as HG test is affected by age, sex, and height of the participants to the study and due to the absence of age and gender stratified normative data for the Italian population, the following strategy was adopted to identify subjects with a good performance to this test: Firstly, a multiple regression model was used to adjust the crude HG scores for age, gender, and height. The standardized residuals of this model were used as ‘adjusted’ scores; secondly, to compare the obtained methylation profiles with respect to the hand grip performance, we stratified our sample according to the top‐quartile of the frequency distribution of the relevant adjusted scores. Following this procedure, we then compared the methylation profiles between subjects with a good performance to the HG test (top‐quartile, Q4) with respect to the profiles obtained for the subjects belonging to the lowest HG quartiles (Q1‐Q3).

Kaplan–Meier estimates were used to obtain the survival curves according to determined methylation levels. The obtained survival curves were then compared using the log‐rank test. As the obtained mortality estimates could be biased by gender and age at inclusion, Cox proportional hazard models were fitted to take into account the possible effects of such confounders on mortality risk. Adjustment for multiple comparisons has been carried out using the Bonferroni procedure (α = 0.05/number of tests). spss v.20 software (SPSS Inc., Chicago, IL, USA) was used for statistical analyses.

## Funding

This work was supported by the European Union's Seventh Framework Programme (FP7/2007‐2011, IDEAL Project, grant number 259679) and by Programma Operativo Nazionale (01_00937)—MIUR ‘Modelli sperimentali biotecnologici integrati per lo sviluppo e la selezione di molecole di interesse per la salute dell'uomo’.

## Conflict of interest

The authors declare no competing financial interests.

## Author contributions

PDA, AM, DB, and GP designed the study; PDA performed the experiments; AM performed the statistical analysis; SG, VM, and AC provided the human blood samples belonging to RD1 and RD2; MM provided rat tissues; DB and GP wrote the initial draft; PDA, AM, MM, DB, and GP participated in critical revision and approved the final manuscript before submission.

## Supporting information


**Fig. S1** Human rDNA promoter.
**Fig. S2** Rat rDNA promoter.
**Fig. S3** Heatmaps showing average methylation levels for different aged rats in the analyzed tissues.
