# Supplementary material for: Methylation of the ribosomal RNA gene promoter is associated with aging and age‐related decline
Source: Aging Cell. 2017 Jun 17;16(5):966–75. doi: 10.1111/acel.12603 (PMC5595699; doi:10.1111/acel.12603)
Supplement: Supplementary file 1 — Fig. S1 Human rDNA promoter. Fig. S2 Rat rDNA promoter. Fig. S3 Heatmaps showing average methylation levels for different aged rats in the analyzed tissues. [file ACEL-16-966-s001.docx]

**LEGENDS OF SUPPLEMENTARY FIGURES**

**Figure S1**. Human rDNA promoter. In (A) the nucleotide sequence of the promoter in which the transcription starting point (+1) is reported in bold. Positive (negative) numbers are assigned to nucleotides downstream (upstream) of nucleotide +1. The Upstream Control Element (UCE) is underlined, the core promoter is indicated by a dashed line. CpG sites analyzed are highlighted in red. Transcription factor binding sites are indicated. (B) Graphical representation of the CpG sites and units, located within human rRNA gene, as according to EpiTYPER software.

**Figure S2.** Rat rDNA promoter. In (A) the nucleotide sequence of the promoter in which the transcription starting point (+1) is reported in bold. Positive (negative) numbers are assigned to nucleotides downstream (upstream) of nucleotide +1. The Upstream Control Element (UCE) is underlined, the core promoter is indicated by a dashed line. CpG sites analyzed are highlighted in red. Transcription factor binding sites are indicated. (B) Graphical representation of the CpG sites and units, located within ratrRNA gene, as according to EpiTYPER software.

**Figure S3.** Heatmaps showing average methylation levels for different aged rats in the analyzed tissues.

**SUPPLEMENTAL FIGURES**

**
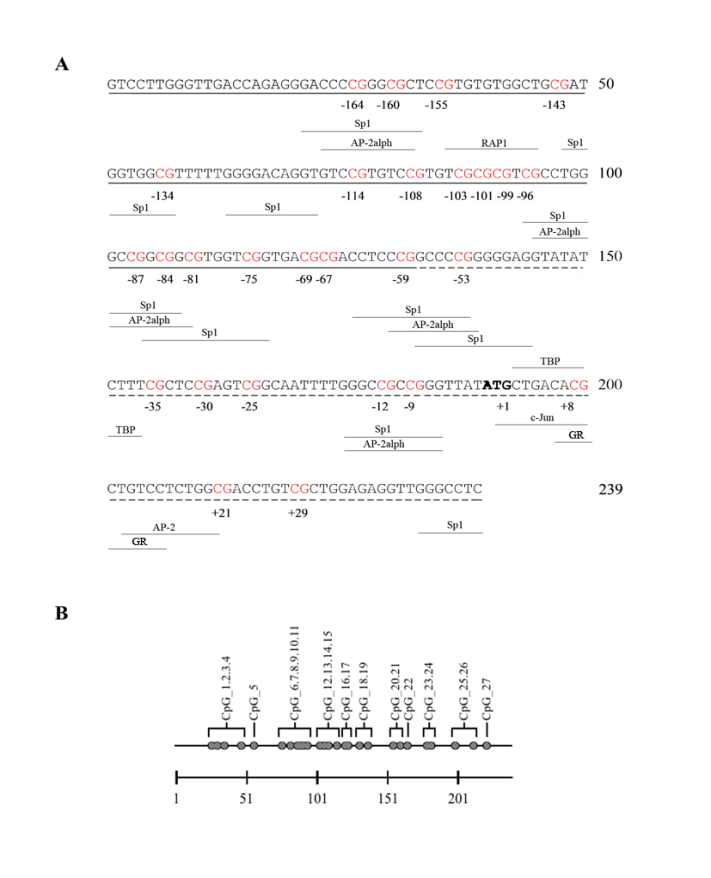
**

**Figure S1**

**
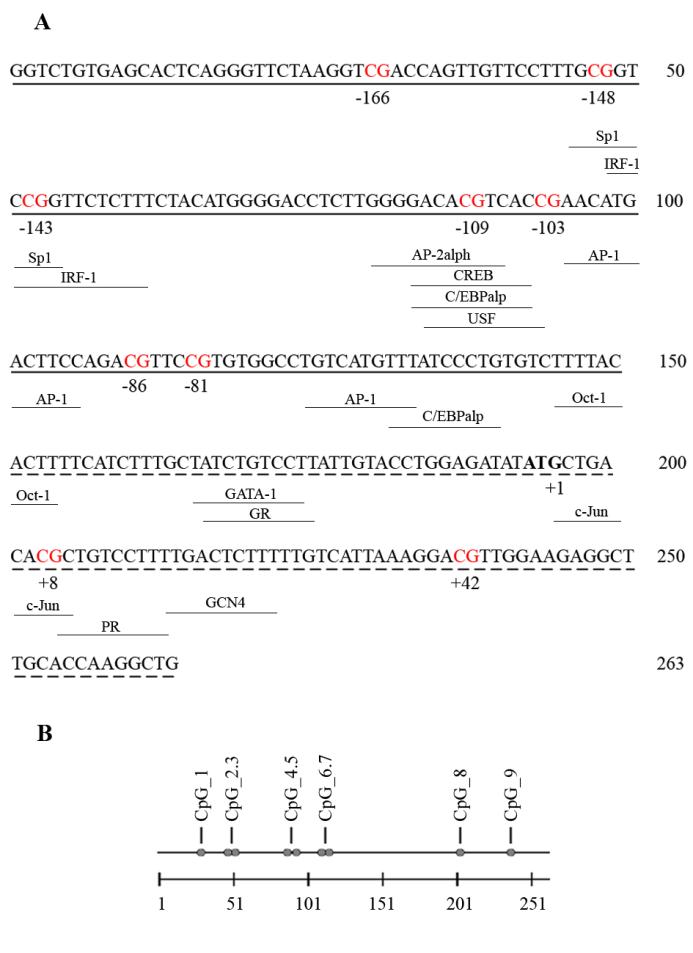
**

**Figure S2**

**
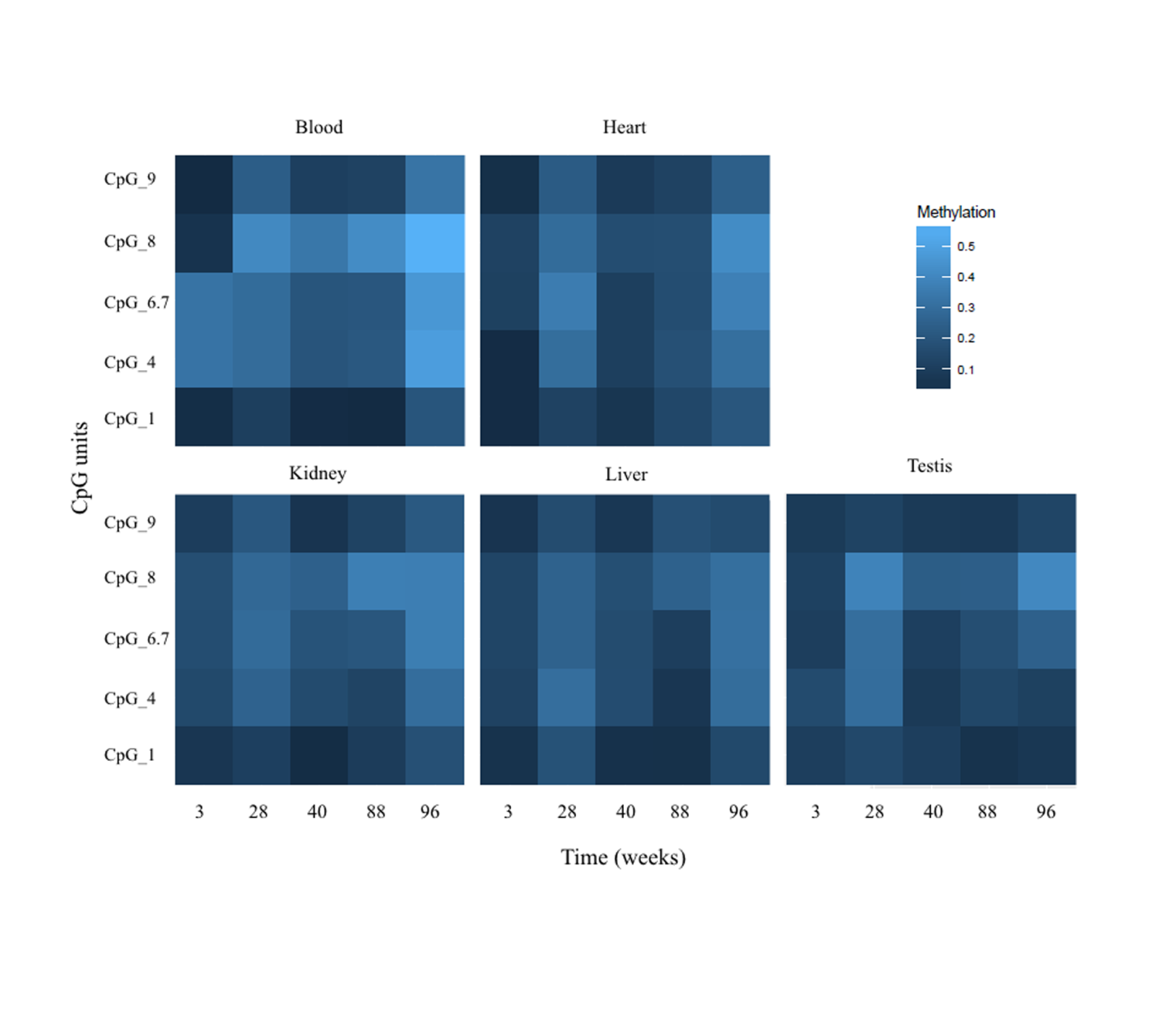
**

**Figure S3**
